# Vascular lipid droplets formed in response to TNF, hypoxia, or OA: biochemical composition and prostacyclin generation

**DOI:** 10.1016/j.jlr.2023.100355

**Published:** 2023-03-17

**Authors:** Marta Z. Pacia, Natalia Chorazy, Magdalena Sternak, Kamila Wojnar-Lason, Stefan Chlopicki

**Affiliations:** 1 Jagiellonian Centre for Experimental Therapeutics (JCET), Jagiellonian University, Krakow, Poland; 2 Doctoral School of Exact and Natural Sciences, Jagiellonian University, Krakow, Poland; 3 Chair of Pharmacology, Jagiellonian University, Krakow, Poland

**Keywords:** endothelium, inflammation, atglistatin, lipolysis, prostacyclin, Raman spectroscopy, fluorescence imaging, angiotensin II, lipid droplets, adipose triglyceride lipase

## Abstract

Biogenesis of lipid droplets (LDs) in various cells plays an important role in various physiological and pathological processes. However, the function of LDs in endothelial physiology and pathology is not well understood. In the present work, we investigated the formation of LDs and prostacyclin (PGI_2_) generation in the vascular tissue of isolated murine aortas following activation by proinflammatory factors: tumor necrosis factor (TNF), lipopolysaccharides (LPS), angiotensin II (AngII), hypoxic conditions, or oleic acid (OA). The abundance, size, and biochemical composition of LDs were characterized based on Raman spectroscopy and fluorescence imaging. We found that blockade of lipolysis by the adipose triglyceride lipase (ATGL) delayed LDs degradation and simultaneously blunted PGI_2_ generation in aorta treated with all tested proinflammatory stimuli. Furthermore, the analysis of Raman spectra of LDs in the isolated vessels stimulated by TNF, LPS, AngII, or hypoxia uncovered that these LDs were all rich in highly unsaturated lipids and had a negligible content of phospholipids and cholesterols. Additionally, by comparing the Raman signature of endothelial LDs under hypoxic or OA-overload conditions in the presence or absence of ATGL inhibitor, atglistatin (Atgl), we show that Atgl does not affect the biochemical composition of LDs. Altogether, independent of whether LDs were induced by pro-inflammatory stimuli, hypoxia, or OA and of whether they were composed of highly unsaturated or less unsaturated lipids, we observed LDs formation invariably associated with ATGL-dependent PGI_2_ generation. In conclusion, vascular LDs formation and ATGL-dependent PGI_2_ generation represent a universal response to vascular proinflammatory insult.

Endothelium covers the innermost layer of blood and lymphatic vessels and fulfills many functions in maintaining cardiovascular homeostasis. The endothelial barrier between the vascular lumen and tissue regulates the vessel diameter and blood fluidity, takes part in the immune system functioning, and regulates the endothelial inflammation (1, 2). Over the course of evolution, endothelial cells (ECs) as the first cells in the vascular lumen subjected to alteration in the blood composition have promoted the development of highly reproducible mechanisms of counteracting disturbances in homeostasis. Depending on the chain of events, the endothelial response to stimulant can be considered as *activating* or *dysfunctional* (3). The functional reaction of the endothelium in response to stress factors such as cytokines, toxins, hypoxia, and so forth should be viewed as a consequence of the physiological response of the endothelium, and then the endothelium is called *activated*. Nevertheless, variable stress factors might result in permanent alterations in endothelial phenotype viewed as *dysfunctional* (3). It is of importance in endothelial biology to delineate the mechanisms that offset endothelial activation and prevent endothelial dysfunction for example in response to endothelial proinflammatory insult.

Endothelial inflammation may be triggered by proinflammatory factors, for example, tumor necrosis factor (TNF), lipopolysaccharides (LPSs), or angiotensin II (AngII). TNF-induced endothelial inflammation is mediated by the TNFR1 and death domain protein (TRADD), which, in turn, binds receptor-interacting protein 1 (RIP1) and TRAF2 forming the TRADD-RIP1-TRAF2 complex, activating proinflammatory endothelial response through the NF-κB pathway (4). Bacterial endotoxin LPS, a major component of the outer membranes of Gram-negative bacteria, acts on the endothelium through the soluble form of CD14 (sCD14) (5) or by TLR-4 receptor and the subsequent activation of nuclear protein complex NF-κB (5). The effects of AngII are mediated by its binding into the angiotensin type 1 (AT1R) or type 2 (AT2R) receptors with opposite actions (6): AT1R is primarily responsible for the prohypertensive and proinflammatory activities of AngII, whereas the AT2R is reported to induce vasoprotective effects. Activation of the AT1R increases the production of reactive oxygen species and activates nuclear factor kappa B and in consequence, inactivates nitric oxide (NO) and reduces its production by uncoupling endothelial NO synthase (6). Hypoxia is able to activate the ECs via activation of hypoxia-inducible factor and thereby initiates a cascade of reactions including diminished secretion of NO and increased production of reactive oxygen species (7, 8). An overload of oleic acid (OA) forces the endothelium to store OA in an esterified form and protects the tissues from excess lipids (9). The common result of the action of aforementioned proinflammatory factors: TNF, LPS, AngII hypoxia, or OA is the activation of vascular endothelium and the subsequent activation of nuclear protein complex NF-κB responsible for the transcription of relevant genes including cyclooxygenase-2 (COX-2) resulting in increased synthesis of eicosanoids (4). As was previously shown (10), the increased generation of prostacyclin (PGI_2_) in activated endothelium was associated with the formation of lipid droplets (LDs).

LDs are spherical cellular organelles rich in triacylglycerols and cholesteryl esters intrinsically related to physiological cellular energy storage and metabolism (11). The main functions of LDs are the regulation of lipids metabolism (12), protein binding, and inactivation, intracellular transport of fats, e.g., to the mitochondria, and intracellular signaling (13). Although the general knowledge on endothelial LDs is growing, neither the precise function of LDs nor the pathway of their biogenesis have been fully revealed. The biogenesis of LDs was recently suggested to represent an integral part of vascular inflammation in TNF-activated isolated vascular wall *ex vivo* (10, 14) or in LPS-activated (15), TNF-activated (16) ECs *in vitro*. It was shown that the formation and degradation of endothelial LDs were regulated by Rac1 and adipose triglyceride lipase (ATGL), respectively, in TNF-stimulated isolated murine aorta (14). Moreover, previous papers showed that LDs act as critical centers of cellular metabolism to buffer increased lipid levels (9). These findings document the active role of LDs in endothelial homeostasis; however, further studies of the characteristics of vascular LDs are needed to reveal the complete pathophysiological role of LDs in the endothelium.

Here, we aimed to better characterize the formation of LDs in response to TNF, LPS, AngII, hypoxia, or OA in ECs within the isolated murine aorta. In particular, in response to each of the stimuli, the abundance, size, and biochemical composition of LDs was characterized as well as ATGL-dependent PGI_2_ generation. The biochemical composition of LDs in this report was analyzed based on Raman imaging. Raman spectroscopy is a label-free and nondestructive technique that could provide valuable information about the biochemical compositions of the biological samples. In particular, this technique was used previously to study endothelium (15, 17), vascular wall in atherosclerosis (18), diabetes (19), or hypertension (20). The spectral characteristics of a given cell type is different (21). Similarly, various type of tissues exhibit distinct patterns of distribution of chemical compounds (22, 23, 24, 25). Due to large Raman scattering cross-section for lipids, bands originating from lipids can be clearly detected in Raman spectra, what enables a reliable detection of lipid structures inside biological samples. Importantly, Raman imaging allows measurements of intracellular lipids without externally labeling them and provides a possibility for the detection, indication of precise localization (endothelium vs. smooth muscle cells), quantitative assessment, and most importantly, the analysis of the biochemical composition of LDs in biological samples including vascular wall (14). In this paper, the specificity of Raman imaging enabled recognition of newly formed LDs in the activated endothelium and their biochemical characterization in response to given proinflammatory stimulus, followed by discrimination of LDs using hierarchical cluster analysis (HCA).

## Materials and methods

All experimental procedures involving animals were conducted according to the Guidelines for Animal Care and Treatment of the European Communities and the Guide for the Care and Use of Laboratory Animals published by the US National Institutes of Health (NIH Publication No. 85-23, revised 1996). All procedures were approved by the second Local Ethical Committee on Animal Experiments. Male C57BL/6J mice (aged 8–12 weeks) were purchased from Mossakowski Medical Research Institute, Polish Academy of Sciences, Warsaw, Poland and were housed in a temperature-controlled environment (22–25°C), 12-h light/day cycle, and unlimited access to standard laboratory diet.

Mice were euthanized by an intraperitoneal injection of a mixture consisting of ketamine and xylazine (100 mg ketamine/10 mg xylazine/kg body weight). The chest (albo chest cavity) was exposed, and the thoracic aorta was dissected. Then, the aorta was cleaned from the surrounding tissue, cut into rings, and transferred into minimal essential medium (Sigma Aldrich) supplemented with 1% minimal essential medium vitamins (Sigma Aldrich), 1% antibiotics (penicillin 10,000 U/ml and streptomycin 10,000 μg/ml and Amphotericin B 25 μg/ml; Thermo Scientific), 1% nonessential amino acids (Sigma Aldrich), and 20% fetal bovine serum (Thermo Scientific). The aorta was incubated in the presence of TNF (10 ng/ml, 24 h, N = 5), LPSs from *Escherichia coli* O111:B4 (10 μg/ml, 24 h, N = 5), AngII (1 μM, 24 h, N = 6), hypoxic condition (5% O_2_, 24 h, N = 6), OA (1 mM, 24 h, N = 6), atglistatin (Atgl; 10 μM, 24 h, N = 6), TNF with Atgl (TNF+Atgl; 10 ng/ml and 10 μM, respectively, 24 h, N = 6), LPSs with Atgl (LPS+Atgl; 10 μg/ml and 10 μM, respectively, 24 h, N = 6), AngII (AngII+Atgl; 1 μM and 10 μM, respectively, 24 h, N = 6), hypoxic condition with Atgl (hypoxia+Atgl; 5% O_2_ and 10 μM, respectively, 24 h, N = 5), OA with Atgl (OA+Atgl; 1 mM and 10 μM, respectively, 24 h, N = 5). The untreated aorta was maintained in the medium for 24 h and was used as a control (N = 6).

### Immunostaining of aorta en face

For immunostaining imaging of aorta en face, the resected and split-open arteries were tightly glued to the Cell-Tak®-coated microscopic glasses and preserved by a 10-min soak in 4% paraformaldehyde. Tissue samples were blocked with TNB blocking buffer (0.1 M Tris-HCl pH 7.5, 0.15 M NaCl, and 0.5% (w/v) blocking reagent; PerkinElmer FP1020) for 3–4 h, and then incubated with CD31 antibody (Abcam, 1:50) diluted in TNB blocking buffer overnight at 4°C. As secondary antibody, Alexa Fluor 647 nm goat-anti-rabbit (Jackson Immuno Research; 3:600) was used at room temperature for 3 h. BODIPY 493/503 diluted in PBS at the final concentration of 0.1 mg/ml was applied for 1 h to delineate LD, and Hoechst 33,258 (Sigma; 1:1000) was used to highlight nuclei. Samples were measured by 40× magnification objective on CQ1 Confocal Quantitative Image Cytometer (Yokogawa).

### Raman imaging of aorta en face

For Raman imaging of aorta en face, the resected and split-open arteries were tightly glued to the Cell-Tak®-coated calcium fluoride surface and preserved by a 10-min soak in 4% buffered formalin. Raman imaging was performed in fluid with a Confocal Raman Imaging system (WITec alpha 300, Ulm Germany) supplied with a UHTS 300 spectrograph (600 grooves·mm^−1^ grating, resolution of 3 cm^−1^) and a CCD detector (Andor, DU401A-BV-352) using a 63× water immersion objective (Nikon Fluor, NA = 1, the Netherlands). A laser excitation wavelength of 532 nm, a laser power of *ca*. 30 mW, and an integration time of 0.4 s per spectrum were used in all measurements. The nominal minimal lateral and depth resolution for our setup is 0.32 and 0.53 μm, respectively, and sampling density of 0.38–0.50 and 0.5–1.0 μm in x/y and z direction, respectively, were used. Depth profiling of the tissue was obtained by multiple imaging of the same line in several layers of the sample. Data matrices were analyzed using WITec Project software (background subtraction using a polynomial of degree 3 and the automatic removal of cosmic rays). The analysis of the spectra was supported by Cluster Analysis (K-means, Manhattan distance, WITec Project Plus). For study of heterogeneity of endothelial LDs within aorta, the single Raman spectra were extracted from the center of each LD and then averaged. The OPUS 7.2 program was used for calculations of the integral intensity of the bands at *ca*. 1660 and 1445 cm^−1^ in the 1733–1767 and 1563–1712 cm^−1^ spectral ranges, respectively. Integration was performed using method D: the integral was defined by the wavenumber limits and the horizontal baseline determined by a chosen baseline point (OPUS 7.2). The single spectra were used for HCA using the Ward’s algorithm in OPUS 7.2 applied to the following spectral regions: 600–3030 cm^−1^. These spectral regions were chosen because of the optimal separation of spectra.

### Immunostaining of cross-section of aorta: expression of intercellular cell adhesion molecule 1

Rings of the aorta were embedded in the OCT medium (Thermo) and frozen at −80°C using Leica CM1920 automatic cryostat (Leica, Wetzlar, Germany). The 5 μm thick cross-section slides were put on the microscopic glasses coated with poli-L-lysine and fixed for 10 min in 4% buffered formalin (Merck) and used for immunostaining of intercellular cell adhesion molecule 1 (ICAM-1). Aortic rings were preincubated with 5% normal goat serum (Jackson Immuno Research) and 2% dry milk in PBS, then immunostained using rat-anti-mouse ICAM-1 (eBioscience; 1:200) primary antibody overnight at 4°C. A secondary antibody Cy3-conjugated goat-anti-rat (Jackson Immuno Research; 1:300) was used, respectively, for 30 min at room temperature. Cell nuclei were visualized by Hoechst 33258 (Sigma; 1:1000) solution, and unspecific fluorescence of aortic elastic fibers were used as a background counterstaining channel. Images were acquired using an AxioCam HRm digital monochromatic camera and an AxioObserver.D1 inverted fluorescent microscope (Carl Zeiss). ICAM-1 fluorescence was calculated as the ICAM-1 expression area divided by the tissue area expressed as a percentage (ImageJ program).

### Measurements of PGI_2_ generation in isolated rings of mice aorta

PGI_2_ generation by aortic rings was quantified on the basis of the formation of 6-keto PGF_1α_, a stable metabolite of PGI_2_ in the supernatant of aortic rings, using 6-keto-PGF_1α_ ELISA kit (Enzo Life Sciences).

### Statistical analysis

Calculations were performed using Origin2019b software, and all data were considered significant if *P* ≤ 0.05. After testing for normal distribution (Shapiro–Wilk test), the two-sample *t* test or one-way ANOVA was performed. All values are given as mean ± SEM.

## Results

### Fluorescence-based detection of LDs in murine aorta in response to TNF, LPS, AngII, hypoxia, or OA: effects of the blockade of ATGL

**Fig. 1:**
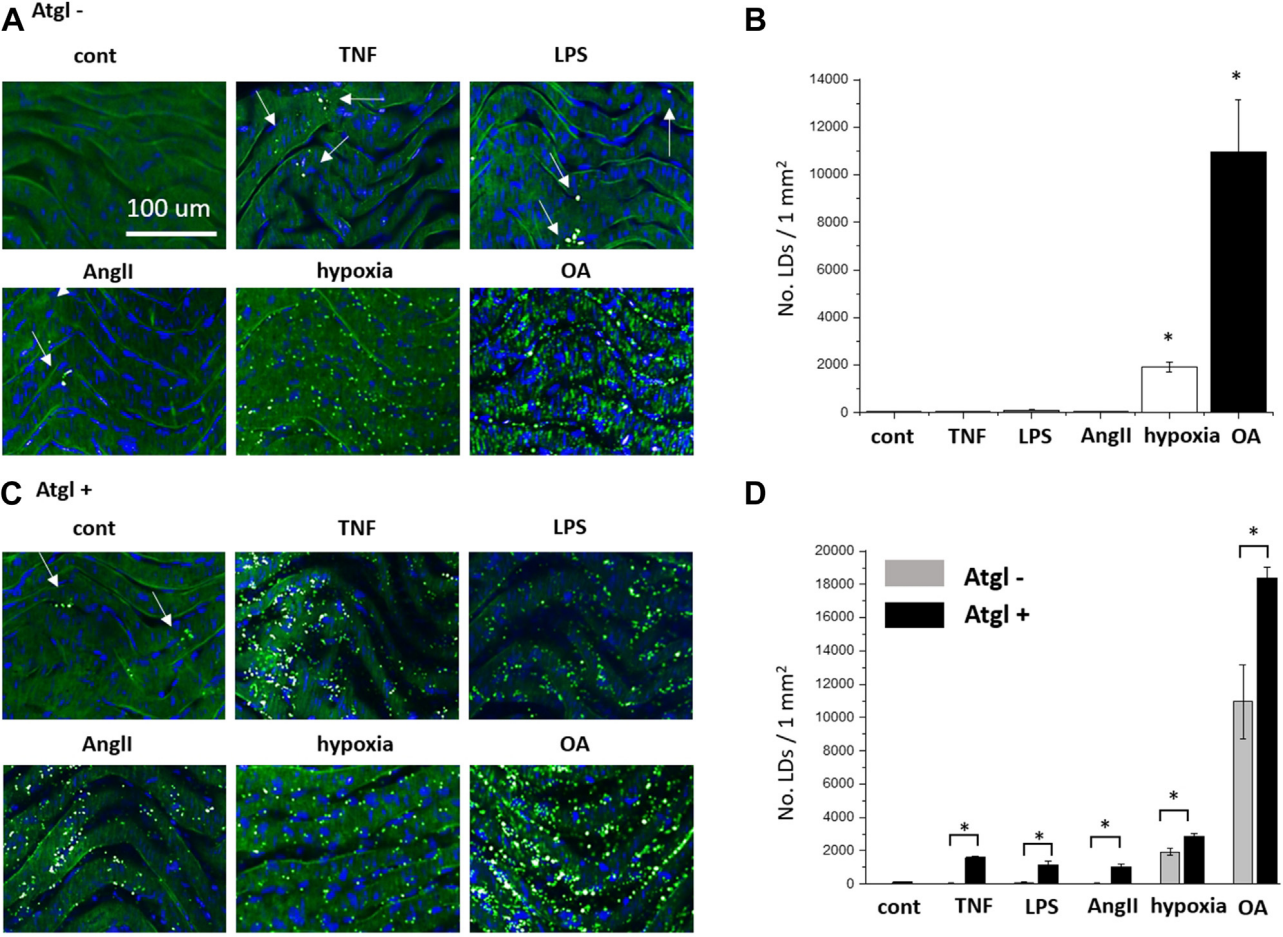
Effects of adipose triglyceride lipase (ATGL) inhibition by atglistatin on number of LDs formed in murine aorta in response to TNF, LPS, Ang II, hypoxia, or OA and analyzed in *en face* aortic preparation. A, B: Fragment of aorta incubated with TNF (10 ng/ml, 24 h), LPS (10 μg/ml, 24 h), or AngII (1 μM, 24 h) showed negligible LDs formation, in contrast to the formation of abundant LDs in isolated blood vessel upon hypoxic condition (5% O_2_, 24 h), or in the presence of OA (1 mM, 24 h). C, D: The ATGL inhibitor (Atgl, 10 μM), abolished LDs degradation in all mentioned-above condition, which results in the observation of LDs in the TNF-, LPS- or AngII-activated aorta, or an increase in the number of LDs in the hypoxic conditions, or in the presence of OA. LDs accumulation was quantified based on BODIPY intensity (green and blue fluorescence originating from LDs and cell nuclei, respectively). ∗*P* < 0.05.

An isolated aorta was stimulated by TNF (10 ng/ml, 24 h), LPS (10 μg/ml, 24 h), AngII (1 μM, 24 h), hypoxic conditions (5% O_2_, 24 h), or by OA (1 mM, 24 h), and LDs were analyzed in *en face* aortic preparation. In aorta stimulated by TNF, LPS, or AngII, LDs were sparsely observed, while in aorta under hypoxic conditions or upon excess of OA, LDs were abundant (Fig. 1A, B). Blockade of the ATGL, a key lipase involved in triacylglycerols hydrolysis by Atgl, (10 μM) resulted in the increased number of LDs in the TNF-, LPS-, or AngII-stimulated aorta in comparison to the aorta treated by TNF, LPS, or AngII in the absence of Atgl (Fig. 1C, D). Atgl alone in nonstimulated aorta did not result in LDs formation. Similarly, in hypoxic condition or in OA-stimulated aorta, the presence of Atgl significantly increased number of LDs. These results suggest that inhibition by Atgl delayed LDs degradation in aorta irrespectively to the stimulus used and whether LDs was of low (TNF, LPS, AngII) or high abundance (hypoxia, OA) (Fig. 1D).

In order to exclude the FBS in the medium as the source of lipids, we have tested the medium without FBS or with 1%, 10% and 20% of FBS (supplemental Fig. S1). In the result, the high-abundance LDs were observed in conditions without FBS. Such data indicate that endogenous rather than exogenous lipids constitute the main source for LDs formation.

### Label-free Raman-based detection of LDs in murine aorta in response to TNF, LPS, AngII, hypoxia, or OA

**Fig. 2:**
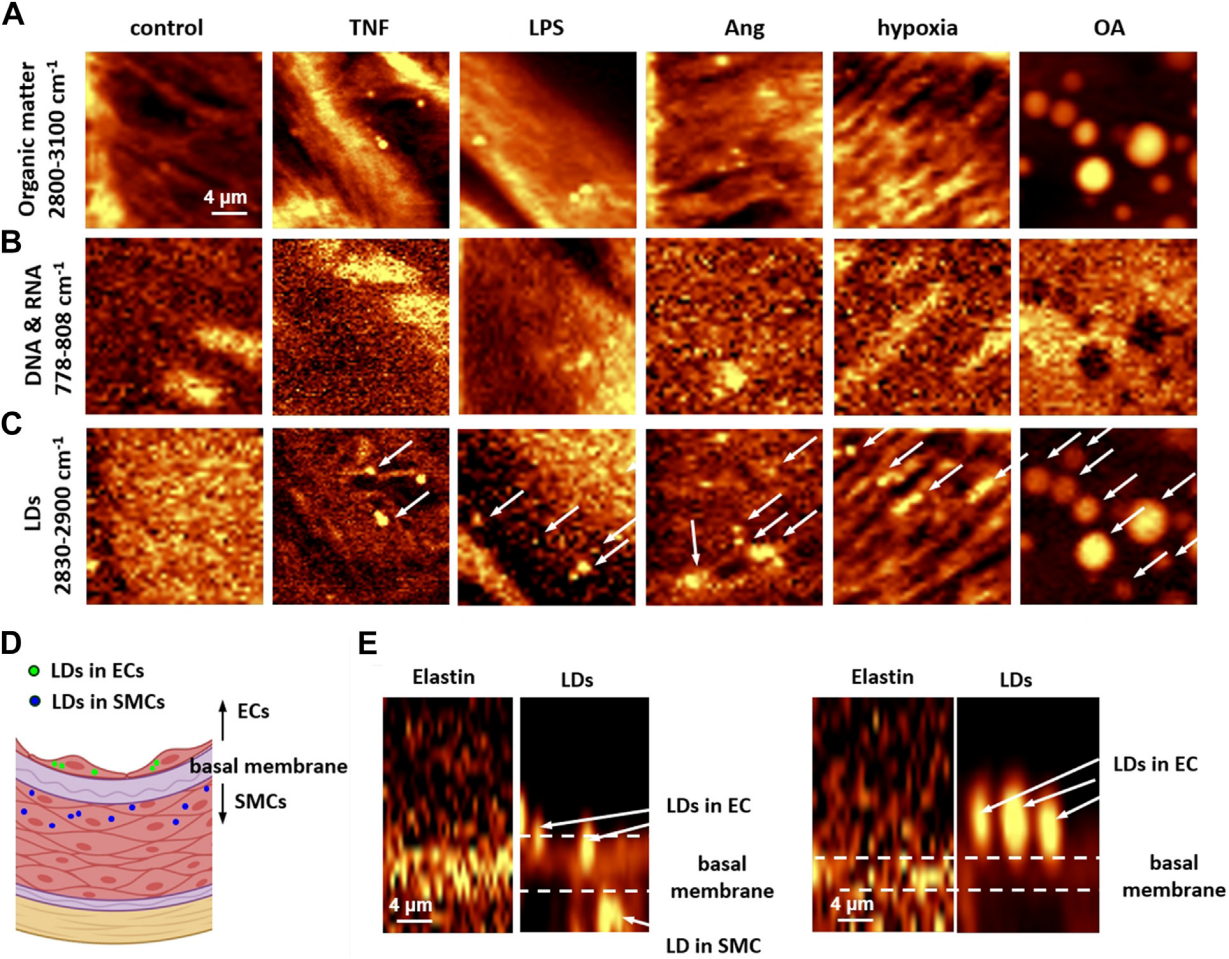
Representative images of biochemical composition of endothelium and endothelial LDs induced by TNF, LPS, AngII, hypoxia, or OA measured by label-free Raman-based confocal imaging in *enface* murine aorta. Raman images showing distribution of (A) organic matter (integration in the 2800–3100 cm^−1^ spectral range), (B) nucleic acids (778–808 cm^−1^), and (C) lipids (2830–2900 cm^−1^) in en face control aorta or aorta activated by TNF (10 ng/ml, 24 h), LPS (10 μg/ml, 24 h), AngII (1 μM, 24 h), hypoxia (5% O_2_, 24 h), or OA (1 mM, 24 h). White arrows indicated LDs. Low-abundance LDs formed in response to TNF, LPS, and AngII were analyzed in the presence of atglistatin (10 μM, 24 h), while high-abundance LDs induced by hypoxia or OA were analyzed in the absence of atglistatin. D: Due to the presence of Raman signal of elastin indicating the localization of the basal membrane, the LDs can be separated into those localized above or below the basal membrane, belonging to endothelial or smooth muscle cells layer, respectively. E: The representative example of depth-profiling Raman measurement for en face aorta after stimulation with LPS was presented; however, analogous measurements were made for other groups of aorta samples activated by various stimuli.

In order to characterize biochemical composition of LDs formed in the isolated aorta stimulated by various stimuli Raman spectroscopy was used. Figure 2 presents two-dimensional Raman images obtained by integrating: the intensity of the C−H stretching vibrations of aliphatic molecules in the 2800–3100 cm^−1^ spectral range, nucleic acids in the 778–808 cm^−1^ range, and C−H stretching vibrations of CH_2_ groups originating mostly from lipids in the 2830–2900 cm^−1^. Representative images (Fig. 2) clearly showed that LDs were visible in the en face aorta after activation by TNF, LPS, AngII, hypoxic condition, or excess of OA (Fig. 2C, *white arrows*). All experiments were performed in the presence of Atgl to be able to detect easily LDs formed in response to stimuli resulting in low abundance of LDs (TNF, LPS, and AngII). During stimulation of the aorta, LDs were formed in both endothelium and smooth muscle cells (Fig. 2D). As the clear-cut border separating the ECs from the smooth muscle cells is the basal membrane made mainly of elastin, we used the Raman signal of elastin and LDs to precisely determine the location of the LDs in endothelium or in smooth muscle cells in the aorta en face preparation. Thus, the Raman depth profiling separated LDs into those localized above or below the basal membrane, belonging to endothelial or smooth muscle cells layer, respectively (Fig. 2D, E).

### Effects of Atgl on biochemical composition and size of endothelial LDs in murine aorta in response to hypoxic condition or excess of OA

**Fig. 3:**
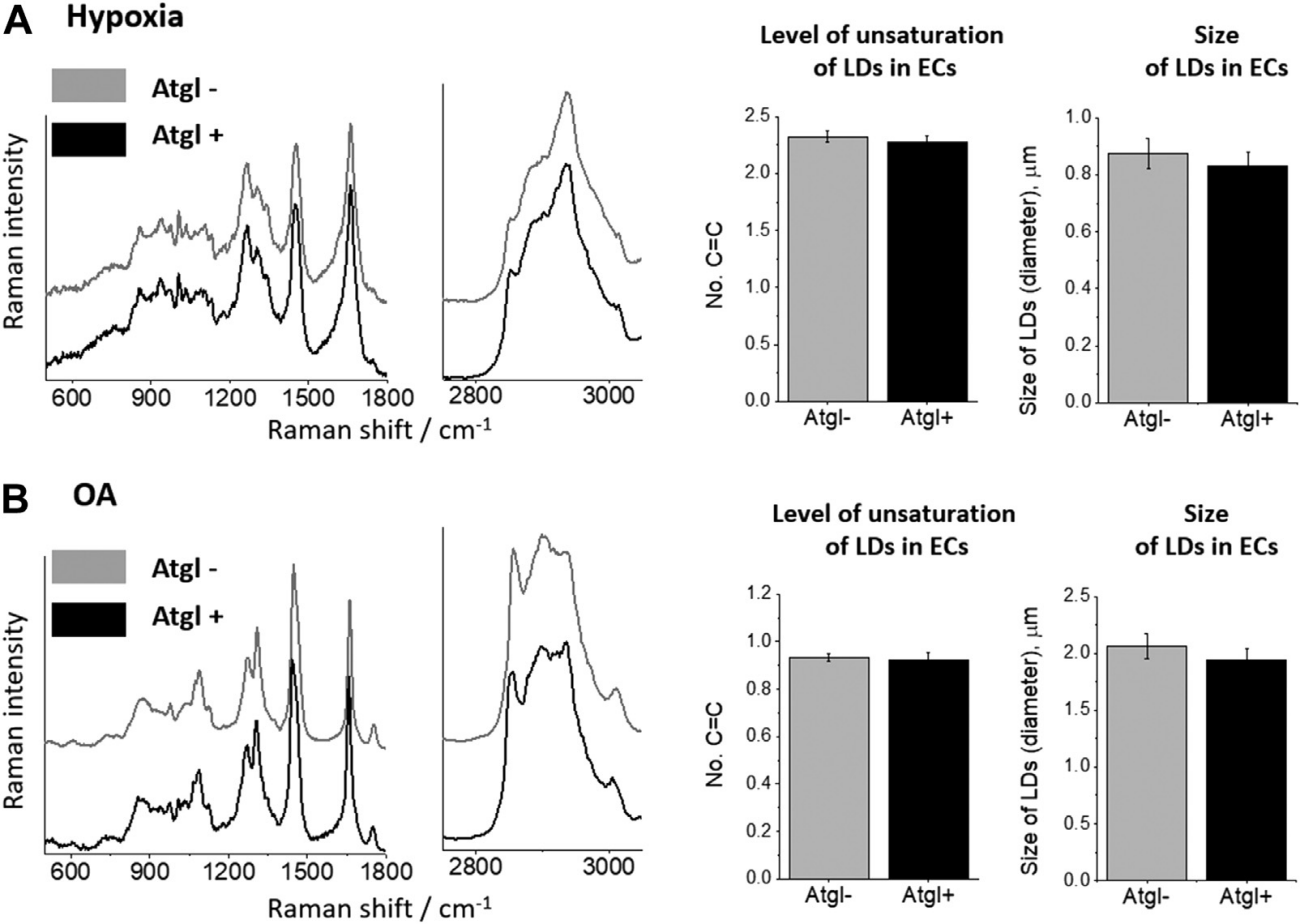
Lack of effect of atglistatin on the biochemical composition of endothelial LDs formed in murine aorta in response to hypoxia or OA. A: Direct comparison of average Raman spectra of endothelial LDs formed under hypoxic conditions (5% O_2_, 24 h) in the presence or absence of Atgl (10 μM, 24 h). B: Similar comparison of average Raman spectra for endothelial LDs in aorta formed in response to the excess of OA (1 mM, 24 h) in the presence or absence of Atgl (10 μM, 24 h). The calculated level of lipids unsaturation (no. C=C), and size of LDs remain unchanged under hypoxic or OA-overload conditions irrespective of the use of atglistatin.

The effect of Atgl on the Raman signature of endothelial LDs was compared for hypoxia- and OA-induced LDs, as these two stimuli induced abundant LDs. A direct comparison of the Raman spectra of endothelial LDs formed under the conditions of hypoxia (Fig. 3A) or in response to OA (Fig. 3B) in the absence and the presence of Atgl revealed that Atgl neither affected the characteristics of the Raman spectrum nor the level of unsaturation of lipids building LDs. The size of LDs was also not affected by Atgl (Fig. 3A, B), suggesting that Atgl affected only the number of LDs (Fig. 1) but no other characteristics of LDs.

### Comparison of biochemical composition of LDs formed in endothelium in response to various stimuli by label-free Raman-based imaging in murine aorta

**Fig. 4:**
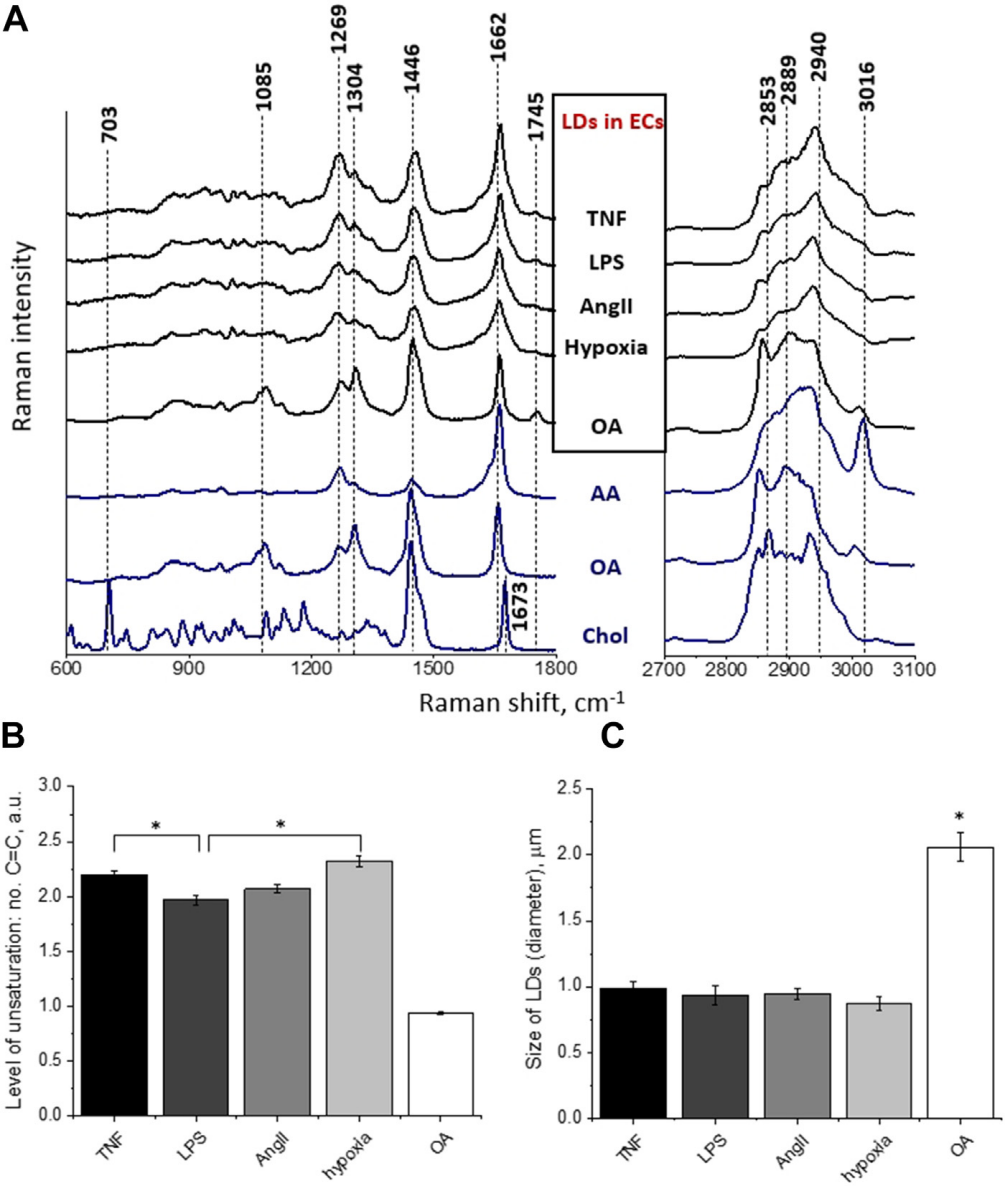
Comparison of chemical characterization of LDs formed in endothelial cells in murine aorta in response to TNF, LPS, Ang II, hypoxia, and OA. A: Average Raman spectra of endothelial LDs formed in aorta treated with TNF (10 ng/ml, 24 h), LPS (10 μg/ml, 24 h), AngII (1 μM, 24 h), hypoxia (5% O_2_, 24 h), or OA (1 mM, 24 h) with comparison to Raman spectrum of arachidonic acid (AA), oleic acid (OA), and cholesterol (Chol). LDs formed in response to all used stimuli were analyzed in the presence of atglistatin (10 μM, 24 h). B: The comparison of the level of unsaturation of lipids in endothelial LDs and (C) size of endothelial LDs in aorta treated with various stimuli and analyzed in *en face* preparation. ∗*P* < 0.05.

To compare the chemical composition of LDs formed in ECs and analyzed in *en face* aorta preparation after stimulation with TNF, LPS, AngII, hypoxia, or OA, the average spectra of LDs were presented and compared to spectra of arachidonic acid (AA), OA, and cholesterol (Chol; Fig. 4). Bands at 1269, 1662, and 3016 cm^−1^, assigned to the in-plane C–H bending, C=C stretching, and =C–H stretching vibrations (26), respectively, indicated the unsaturated character of lipids inside LDs in ECs within *en face* aorta. LDs formed in response to all used stimuli were analyzed in the presence of Atgl (10 μM, 24 h). Features observed at 1300–1310 cm^−1^ range (attributed to the CH_2_/CH_3_ twisting vibrations (26)) were related to lipids in general, as well as bands in the 1410–1500 cm^−1^ range (attributed to the CH_2_ bending vibration (26)). The latter assignment was confirmed by the presence of the band at *ca*. 1745 cm^−1^, attributed to the carbonyl stretching vibrations of cholesteryl esters, phospholipids, and triglycerides (26). The hallmark of LDs was a considerable increase of the components at *ca*. 2853 (due to the CH_2_ stretching vibrations in lipids and fatty acids) and 2889 cm^−1^ (due to the CH_3_ stretching vibrations in lipids and fatty acids) relative to band at 2940 cm^−1^ (resulting in an overall increase of the C-H band integral intensity) (27).

The average spectra of LDs formed in ECs and analyzed in *en face* aorta following the response to TNF, LPS, AngII, or upon hypoxic conditions (Fig. 4A) exhibited a number of similar features. All mentioned spectra did not express distinct band at *ca*. 703 cm^−1^ which suggested the negligible content of chol within LDs, and all the spectra contained band at *ca*. 1745 cm^−1^, attributed to the carbonyl stretching vibrations indicating that the main component of LDs were triacylglycerols, not free fatty acids. Moreover, the level of unsaturation of lipids in endothelial LDs was determined as the n(C=C)/n(CH_2_) intensity ratio obtained by integration of the respective Raman bands (1662/1446 cm^–1^; Fig. 4B). Results showed slightly lower average level of unsaturation of LDs in ECs activated by LPS (1.97 ± 0.05) in comparison to TNF (2.20 ± 0.04) or hypoxia (2.3 ± 0.05). Simultaneously, no statistically significant differences were observed between the level of unsaturation in LDs in AngII- (2.1 ± 0.03), TNF- or hypoxia-treated aorta.

Endothelial LDs formed in the response to OA were definitely more saturated (average number of C=C bonds equaled 0.93 ± 0.02) than LDs formed in the response to TNF, LPS, AngII, or hypoxia, and theirs Raman signature resembled mainly glyceryl trioleate (Fig. 4A, B). Except for the statistically significantly larger LDs during incubation with OA (2.1 ± 0.11), the remaining LDs observed in the response to TNF, LPS, AngII, or hypoxia were of a similar size: 0.93 ± 0.07, 2.3 ± 0.05, 0.95 ± 0.04, and 0.99 ± 0.05, respectively (Fig. 4C).

**Fig. 5:**
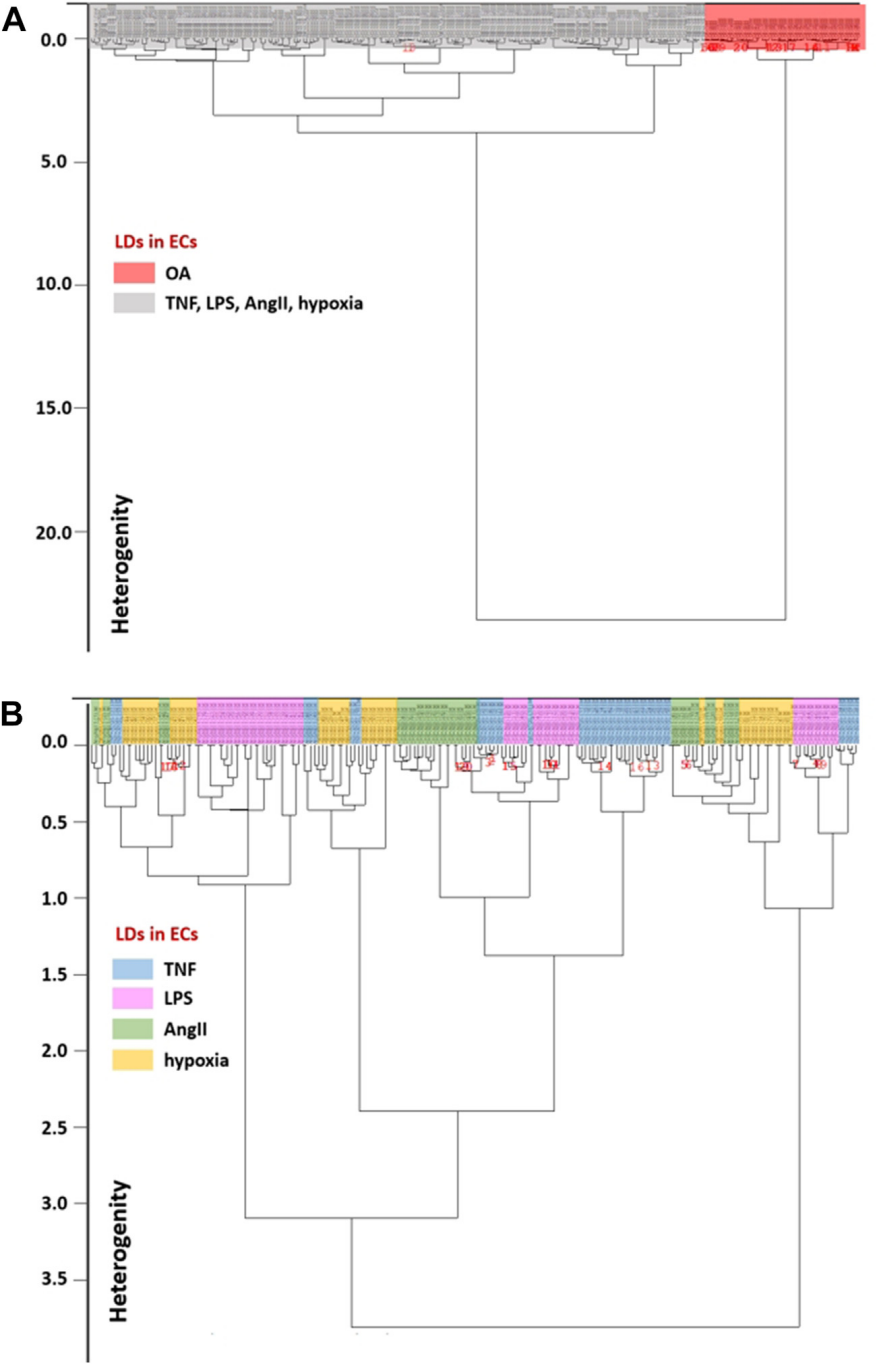
Classification of single spectra of endothelial LDs formed in response to TNF, LPS, AngII hypoxia, or OA assessed by HCA. A: HCA of Raman spectra separated the spectra of LDs from endothelium stimulated by the excess of OA (red color) but (B) did not allow to distinguish LDs in endothelium stimulated by TNF, LPS, AngII, or hypoxia (blue, pink, green, and yellow, respectively). LDs formed in response to all used stimuli were analyzed in the presence of atglistatin (10 μM, 24 h). Atgl alone did not altered biochemical features of LDs, only their size and number (see text for details).

HCA was applied to obtain classification of single spectra of LDs from endothelium stimulated by TNF, LPS, AngII, hypoxia, or OA according to their heterogeneity. The best discrimination, with 100% accuracy, was obtained between Raman spectra of LDs in en face aorta stimulated by OA as compared with other factors (Fig. 5A; red and gray color-coding for LDs formed in the response to OA and other factors, respectively). HCA of Raman spectra did not allow to distinguish biochemical composition of LDs formed in endothelium in response to TNF, LPS, AngII, or hypoxia (Fig. 5B). The Raman spectra of LDs from endothelium stimulated by TNF, LPS, AngII, or hypoxia were mixed in HCA, which indicated that the existing differences in biochemical composition were too small to be separated by HCA classification.

### Effects of TNF, LPS, AngII, hypoxia, or OA on expression of ICAM-1 in ECs in isolated murine aorta

**Fig. 6:**
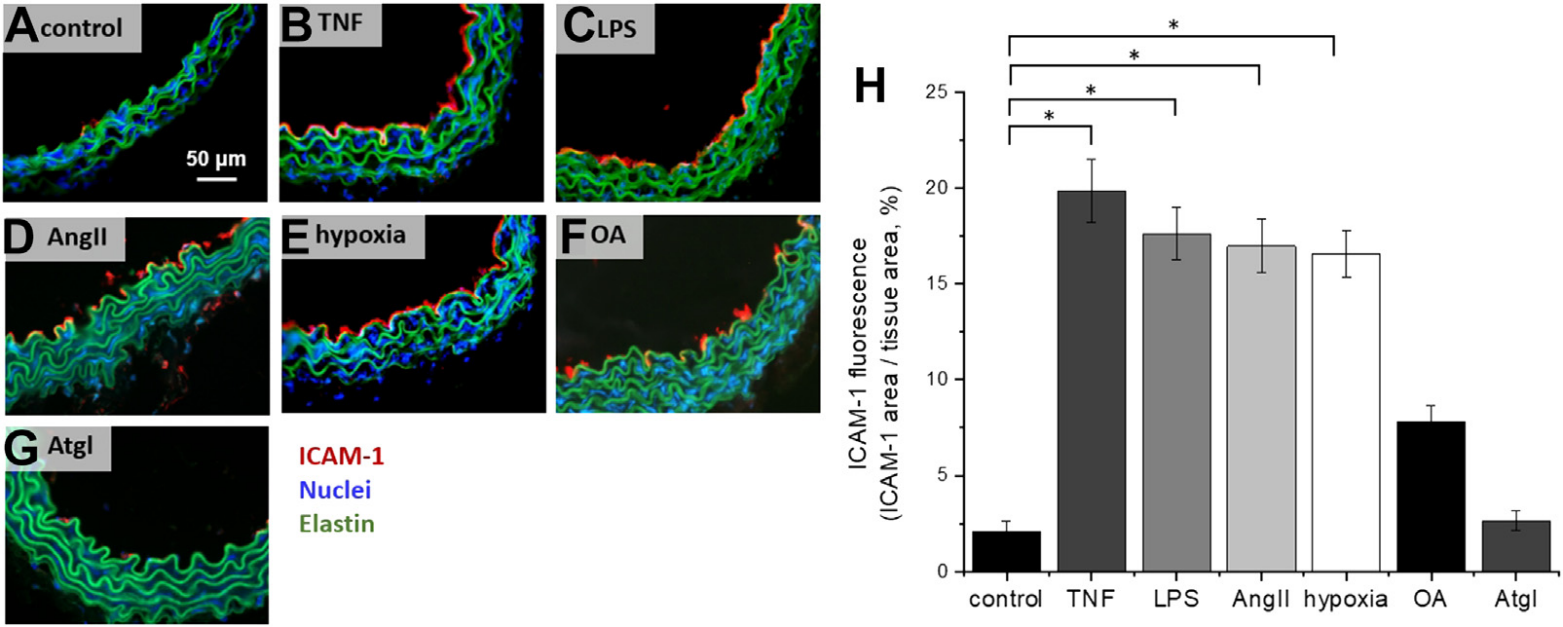
Effects of TNF, LPS, AngII, hypoxia, or OA on expression of ICAM-1 in ECs in isolated murine aorta. Microphotographs of ICAM-1 expression in cross-section of (A) control aorta, aorta treated with (B) TNF (10 ng/ml, 24 h), (C) LPS (10 μg/ml, 24 h), (D) AngII (1 μM, 24 h), (E) hypoxic condition (5% O_2_, 24 h), (F) OA (1 mM, 24 h), or (G) Atgl (10 μM, 24 h). Red, blue, and green fluorescence originate from ICAM-1, cell nuclei, and elastin fibers, respectively. H: Quantification of ICAM-1 expression of studied groups of samples. ∗*P* < 0.05.

To verify whether in our experimental conditions various stimuli used caused the endothelial inflammation, the immunohistochemical staining and fluorescence imaging of the ICAM-1 was visualized and quantified (Fig. 6A–H). The images of ICAM-1-stained TNF-, LPS-, AngII-, or hypoxia-activated ECs confirmed the overexpression of ICAM-1 in comparison to the control, which confirmed the development of endothelial inflammation by all stimuli used (Fig. 6H). Excess of OA induced increased expression of ICAM-1 that however did not reach the statistical significance. Atgl alone was without an effect on ICAM-1 expression (Fig. 6H).

### Effects of TNF, LPS, AngII, hypoxia, or OA on PGI_2_ generation in murine aorta: effects of the blockade of ATGL

**Fig. 7:**
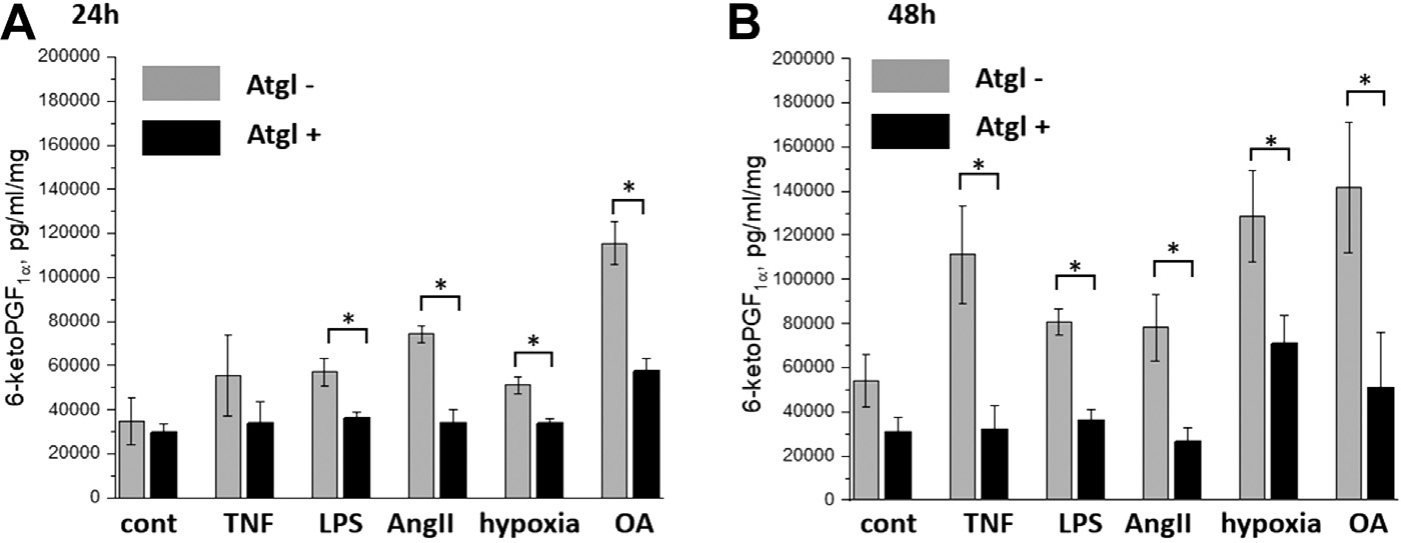
Effects of atglistatin on vascular PGI_2_ generation induced by TNF, LPS, AngII, hypoxia, or OA. Prostacyclin generation measured based on its stable metabolite: 6-keto-PGF1α after (A) 24 or (B) 48 h in unstimulated aorta or aorta activated by TNF (10 ng/ml), LPS (10 μg/ml), AngII (1 μM), hypoxia (5% O_2_), or OA (1 mM). In the presence of atglistatin, 6-keto-PGF1α levels were lowered irrespectively of the stimulus used. ∗*P* < 0.05.

As shown in Figure 7, TNF, LPS, AngII, hypoxia, or OA, all these stimuli resulted in an increased generation of PGI_2_ in aorta samples (measured based on its stable metabolite - 6-keto-PGF_1α_) after 24 or 48 h of incubation, though only for some among the stimuli, the difference reached statistical significance. Importantly, in the presence of Atgl, generation of PGI_2_ induced by LPS, AngII, hypoxia, or OA after 24 h incubation (Fig. 7A) and induced by TNF, LPS, AngII, hypoxia, or OA after 48 h incubation (Fig. 7B) was significantly reduced as compared to aorta without Atgl suggesting ATGL-dependent PGI_2_ generation.

## Discussion

In the present work, we demonstrated that proinflammatory activation of ECs in an isolated aorta using variety of stimuli such as TNF, LPS, AngII, hypoxia, or OA led to the formation of LDs and concomitant PGI_2_ generation. The inhibition of ATGL increased number of endothelial LDs and simultaneously abrogated PGI_2_ generation suggesting a key role of ATGL in dynamic formation of LDs in vascular inflammation and in the generation of PGI_2_. Importantly, biochemical composition of LDs as defined by Raman imaging was not uniform for all proinflammatory stimuli, but the formation of LDs was invariably associated with PGI_2_ generation. Altogether, our results suggest that LDs formation and ATGL-dependent PGI_2_ generation represent a universal responses to vascular proinflammatory insult.

LDs formation in endothelium was previously demonstrated in cultured ECs in response to TNF (16), LPS (15), or OA (9, 28). In contrast to the isolated aorta preparation, LDs formed in cultured cells under the influence of proinflammatory factors were stable and did not require the use of Atgl (15, 16), which indicates a difference in the formation and degradation of LDs in cell line as compared with the isolated blood vessel. These results seem to suggest that dynamics of LDs in vascular wall involve the interactions between ECs and smooth muscle cells and possibly other cells naturally occurring in the tissue. The endothelium in the isolated blood vessel retains its phenotypic characteristics of *in vivo* situation better than ECs in culture. Therefore, research conducted on fragments of the vessel wall may better mimic the *in vivo* condition, as regards LDs formation in the aorta in vascular inflammation as compared with ECs in culture. However, obviously, isolated vessel is still far from the in vivo system, with blood flow, intercellular communication, and the presence of circulating cells (WBCs, RBCs, etc.) that could all affect composition and formation of LDs.

In the present work, we thoroughly characterized LDs in ECs in isolated aorta and extended the knowledge about vascular ATGL-dependent LDs formation and ATGL-dependent PGI_2_ generation in response to vascular proinflammatory insult. The development of endothelial inflammation was confirmed by the overexpression of ICAM-1 in endothelium (Fig. 6). Dynamic formation and decomposition of LDs in isolated aorta incubated with: TNF, LPS, or AngII (Fig. 1A, B) was demonstrated by showing that blockade of the ATGL resulted on elevated number of LDs in stimulated aorta in comparison to nontreated tissues. However, the scale and the significance of the phenomenon were not uniform. After aorta stimulation by TNF, LPS, or AngII, blockade of ATGL enables LDs observation, while under hypoxia or excess of OA, the action of Atgl was not necessary for the observation of LDs but increases their number (Fig. 1C, D). In the case of hypoxia, it should be considered that the inhibition of ATGL causes the reduction of fatty acids oxidation and fatty acids accumulation in the form of LDs, even though, the literature previously showed that fatty acids are not the main substrate of mitochondrial respiration in the endothelium. Also, data from our laboratory showed that there is no accumulation of LDs during the blockage of mitochondrial respiration (antimycin and rotenone, 5% O_2_, 24 h) compared to control (5% O_2_, 24 h, data not shown). Thus, the increased number of LDs in hypoxia resulted from the blockage of their decomposition and not from reduced oxidation of fatty acids.

These results stay in line with previous studies, where Atgl enhanced LDs accumulation in OA-treated ECs in culture and in aorta (9) and extend them by showing that in the isolated aorta ATGL–dependent degradation of LDs is shared for all the stimuli inducing LD and used here.

Spectroscopic characterization of inflammation-induced LDs in ECs within *en face* aorta releveled the heterogeneity in the biochemical composition of LDs. Raman signature of endothelial LDs under hypoxic or OA conditions in the presence or absence of Atgl revealed that Atgl did not affect the biochemical composition of LDs (Fig. 3), which allows us to believe that Atgl alone had no effect on the biochemical composition of LDs under stimulation conditions where Atgl was necessary for the observation of LDs. The analysis of Raman spectra of LDs in the isolated vessels stimulated by TNF, LPS, AngII, or hypoxia uncovered that they were all rich in highly unsaturated lipids and had a negligible content of phospholipids and chol (Fig. 4A, B), which is consistent with previously analyzed endothelial LDs in TNF-treated aorta (14). A similar Raman signature of LDs from endothelium stimulated by LPS, AngII, TNF, or hypoxia was confirmed by the lack of significant differences in the classification using hierarchical cluster analysis. The best discrimination, with 100% accuracy, was obtained for Raman spectra of LDs formed in response to OA as compared with other factors (Fig. 5B) suggesting a clearly distinct biochemical composition of LD induced by OA as compared with LDs induced by other factors.

Although the presented results clearly show the potential of Raman spectroscopy for the detection, indication of precise localization (endothelium vs. smooth muscle cells [SMCs]), quantitative assessment and the biochemical composition of LDs in biological samples, this technique is not without limitations. Imaging of LDs was carried out in an isolated blood vessel maintaining the original endothelial environment giving a possibility to distinguish vascular LDs formed in endothelium and LDs formed smooth muscle cells. In our study, the Raman signature of the elastin spectrum (which can be treated as the boundary between the endothelium and the SMC) was used to determine the precise location of the LDs and on the other hand overlapped with the Raman signal of the LDs located in the SMC layer. As the consequence, the limitation was that collected Raman signal from LDs in SMC did not allow to analyze the degree of unsaturation of lipids building LDs in SMC (14). Of note, Raman-based analysis was determined on the basis of the intensity ratio of the bands representing an average value of the degree of lipid unsaturation. Thus, without additional mathematical modeling, it was not possible to estimate the percentages of individual lipids class found in the LDs (28) that would require lipidomic-based approach (29). Despite the limitations, the RI technique proved to be great for screening the biochemical composition of LDs under various stimulation conditions and defining differences or similarities between them.

We have noticed the significantly lower degree of unsaturation of lipids building the endothelial LDs formed in aorta in response to OA in comparison to LDs in TNF-, LPS-, AngII-, or hypoxia-stimulated ECs. The lower degree of unsaturation of lipids building the endothelial LDs formed in aorta in response to OA was due to the uptake of fatty acid in the medium, where the endothelium acts as a buffer of excess acids present in the environment and protects the tissues from excess lipids (9). However, despite an excess of OA, endothelial LDs in the OA-stimulated aorta contained a mixture of OA and AA amounted to 91.3% and 8.70%, respectively (28). These findings supported the notion that the formation of LDs even in response to OA is related to the eicosanoids biosynthesis in vascular wall, similar to the well-known role of LDs in the generation of eicosanoids in leukocytes (30, 31). Proinflammatory activation of ECs causes the release of AA from the plasma membrane, the main source of eicosanoids generation via COX-2 or COX-1 pathway (32). However, the remaining free AA can be reacylated to phospholipids or triacyclglycerols (32), and according to our results stored back in LDs (10, 28). The presence of AA in LDs could be indeed a reason for a high degree of lipid unsaturation observed in endothelial LDs in the aorta stimulated by TNF, LPS, AngII, or hypoxic condition (Fig. 4).

The important finding of this work to show that despite the distinct biochemical composition of LDs induced by various stimuli formation of vascular LDs was invariably linked to the generation of PGI_2_. Furthermore, we demonstrated that LDs formation (Fig. 1D) and PGI_2_ generation (Fig. 7), both responses were ATGL-dependent, which highlighted a key role of ATGL in lipids/eicosanoid-synthesizing machinery in vascular wall during inflammation. The function of LDs in the generation of eicosanoids was shown previously in leukocytes (30), also in response to proinflammatory stimulation of leukocytes, including for example cys-LTs (33). Therefore, endothelial LDs also may represent a reservoir of reserve AA, which is released according to the needs of the cell and may be directed metabolized to prostaglandins or alternatively reincorporated to the phospholipids pool. The key enzyme regulating the decomposition of triacylglycerols from LDs and thus indirectly releasing AA, is ATGL (34). ATGL inhibition blocks the decomposition of LDs, thus depriving the cell of the source of AA from LDs, and in the consequence reducing the availability of substrates for the production of eicosanoids. This explains the dependence of PGI_2_ generation on LDs formation, and the factor connecting both phenomena was ATGL.

The generation of vascular PGI_2_ is tightly regulated to ensure a sufficient PGI_2_ level for vasoprotection (35) and classical mechanisms of PGI_2_ generation from vascular wall to afford antiplatelet, antiinflammatory, and antiatherosclerotic activity (36). Here, we linked the increased generation of PGI_2_ in vascular wall to LDs formation induced by proinflammatory factors and to ATGL-dependent pathway. These results suggest the importance of ATGL activity not only for lipolysis but also for vasoprotection. Interestingly, reduced expression of ATGL was shown to enhance TNF-induced ICAM-1 expression in human aortic ECs *via* protein kinase C-dependent activation of NF-κB (37), while global ATGL deficiency leads to pronounced vascular endothelial dysfunction in mice; however, in human subjects, vascular symptoms were not reported for patients with vascular endothelial dysfunction (38). It remains to be established whether vasoprotective mechanism of endogenous PGI_2_ in acute inflammation of the liver (39) or activated by exogenous 1-methylnicotinamide (40, 41) or inhibitors of angiotensin converting enzyme (42) are also related to vascular LDs.

In conclusion, we characterized the formation of endothelial LDs associated with ATGL-dependent PGI_2_ generation in endothelium in isolated murine aorta in response to TNF, LPS, AngII, hypoxia, or OA. The inhibition of ATGL delayed LDs degradation in aorta irrespectively to the stimulus used and whether LDs was of low (TNF, LPS, AngII) or high abundance (hypoxia, OA). Biochemical composition of LDs as defined by Raman imaging was not uniform for all proinflammatory stimuli used, but the formation of endothelial LDs was invariably associated with ATGL–dependent PGI_2_ generation.

## Data availability

All data are contained within the article. Any or additional data are available from the corresponding authors upon reasonable request.

## Supplemental data

This article contains supplemental data.

## Conflicts of interest

The authors declare that they have no conflicts of interest with the contents of this article.

## References

[1] Aird W.C., Laubichler M. (2007). **Endothelial Biomedicine**.

[2] Lüscher T.F., Barton M. (1997). Biology of the endothelium. Clin. Cardiol..

[3] Ten V.S., Pinsky D.J. (2002). Endothelial response to hypoxia: physiologic adaptation and pathologic dysfunction. Curr. Opin. Crit. Care.

[4] Pober J.S., Sessa W.C. (2007). Evolving functions of endothelial cells in inflammation. Nat. Rev. Immunol..

[5] Frey E., Miller D., Jahr T.G., Sundan A., Bazil V., Espevik T. (1992). Soluble CD14 participates in the response of cells to lipopolysaccharide. J. Exp. Med..

[6] Kellici T.F., Tzakos A.G., Mavromoustakos T. (2015). Rational drug design and synthesis of molecules targeting the angiotensin II type 1 and type 2 receptors. Molecules.

[7] Michiels C., Arnould T., Remacle J. (2000). Endothelial cell responses to hypoxia: initiation of a cascade of cellular interactions. Biochim. Biophys. Acta.

[8] Paternotte E., Gaucher C., Labrude P., Stoltz J.-F., Menu P. (2008). Behaviour of endothelial cells faced with hypoxia. Biomed. Mater. Eng..

[9] Kuo A., Lee M.Y., Sessa W.C. (2017). Lipid droplet biogenesis and function in the endothelium. Circ. Res..

[10] Pacia M.Z., Sternak M., Mateuszuk L., Stojak M., Kaczor A., Chlopicki S. (2020). Heterogeneity of chemical composition of lipid droplets in endothelial inflammation and apoptosis. Biochim. Biophys. Acta Mol. Cell Res..

[11] Coleman R.A. (2020). The “discovery” of lipid droplets: a brief history of organelles hidden in plain sight. Biochim. Biophys. Acta Mol. Cell Biol. Lipids.

[12] Ducharme N.A., Bickel P.E. (2008). Minireview: lipid droplets in lipogenesis and lipolysis. Endocrinology.

[13] Arrese E.L., Saudale F.Z., Soulages J.L. (2014). Lipid droplets as signaling platforms linking metabolic and cellular functions. Lipid Insights.

[14] Pacia M.Z., Chorazy N., Sternak M., Fels B., Pacia M., Kepczynski M. (2022). Rac1 regulates lipid droplets formation, nanomechanical, and nanostructural changes induced by TNF in vascular endothelium in the isolated murine aorta. Cell. Mol. Life Sci..

[15] Czamara K., Stojak M., Pacia M.Z., Zieba A., Baranska M., Chlopicki S. (2021). Lipid droplets formation represents an integral component of endothelial inflammation induced by LPS. Cells.

[16] Czamara K., Majzner K., Selmi A., Baranska M., Ozaki Y., Kaczor A. (2017). Unsaturated lipid bodies as a hallmark of inflammation studied by Raman 2D and 3D microscopy. Sci. Rep..

[17] Czamara K., Adamczyk A., Stojak M., Radwan B., Baranska M. (2021). Astaxanthin as a new Raman probe for biosensing of specific subcellular lipidic structures: can we detect lipids in cells under resonance conditions?. Cell. Mol. Life Sci..

[18] Maase M., Rygula A., Pacia M.Z., Proniewski B., Mateuszuk L., Sternak M. (2019). Combined Raman-and AFM-based detection of biochemical and nanomechanical features of endothelial dysfunction in aorta isolated from ApoE/LDLR−/− mice. Nanomedicine.

[19] Pilarczyk M., Mateuszuk L., Rygula A., Kepczynski M., Chlopicki S., Baranska M. (2014). Endothelium in spots–high-content imaging of lipid rafts clusters in db/db mice. PLoS One.

[20] Pacia M., Mateuszuk L., Chlopicki S., Baranska M., Kaczor A. (2015). Biochemical changes of the endothelium in the murine model of NO-deficient hypertension. Analyst.

[21] Kochan K., Kus E., Filipek A., Szafranska K., Chlopicki S., Baranska M. (2017). Label-free spectroscopic characterization of live liver sinusoidal endothelial cells (LSECs) isolated from the murine liver. Analyst.

[22] Majka Z., Czamara K., Janus J., Kępczyński M., Kaczor A. (2022). Prominent hypertrophy of perivascular adipocytes due to short-term high fat diet. Biochim. Biophys. Acta Mol. Basis Dis..

[23] Pacia M.Z., Buczek E., Blazejczyk A., Gregorius A., Wietrzyk J., Chlopicki S. (2016). 3D Raman imaging of systemic endothelial dysfunction in the murine model of metastatic breast cancer. Anal. Bioanal. Chem..

[24] Pacia M.Z., Czamara K., Zebala M., Kus E., Chlopicki S., Kaczor A. (2018). Rapid diagnostics of liver steatosis by Raman spectroscopy via fiber optic probe: a pilot study. Analyst.

[25] Stanek E., Pacia M.Z., Kaczor A., Czamara K. (2022). The distinct phenotype of primary adipocytes and adipocytes derived from stem cells of white adipose tissue as assessed by Raman and fluorescence imaging. Cell. Mol. Life Sci..

[26] Czamara K., Majzner K., Pacia M.Z., Kochan K., Kaczor A., Baranska M. (2015). Raman spectroscopy of lipids: a review. J. Raman Spectra..

[27] Movasaghi Z., Rehman S., Rehman I.U. (2007). Raman spectroscopy of biological tissues. Appl. Spectrosc. Rev..

[28] Pacia M.Z., Majzner K., Czamara K., Sternak M., Chlopicki S., Baranska M. (2020). Estimation of the content of lipids composing endothelial lipid droplets based on Raman imaging. Biochim. Biophys. Acta Mol. Cell Biol. Lipids.

[29] Chitraju C., Trötzmüller M., Hartler J., Wolinski H., Thallinger G.G., Lass A. (2012). Lipidomic analysis of lipid droplets from murine hepatocytes reveals distinct signatures for nutritional stress. J. Lipid Res..

[30] Bozza P.T., Magalhães K.G., Weller P.F. (2009). Leukocyte lipid bodies—biogenesis and functions in inflammation. Biochim. Biophys. Acta Mol. Cell Biol. Lipids.

[31] Weller P.F. (2016). Leukocyte lipid bodies—structure and function as “eicosasomes”. Trans. Am. Clin. Climatol. Assoc..

[32] Triggiani M., Marone G. (1994). Differential roles for triglyceride and phospholipid pools of arachidonic acid in human lung macrophages. J. Immunol. Res..

[33] Bozza P., Bakker-Abreu I., Navarro-Xavier R., Bandeira-Melo C. (2011). Lipid body function in eicosanoid synthesis: an update. Prostaglandins Leukot. Essent. Fatty Acids.

[34] Riederer M., Lechleitner M., Köfeler H., Frank S. (2017). Reduced expression of adipose triglyceride lipase decreases arachidonic acid release and prostacyclin secretion in human aortic endothelial cells. Arch. Physiol. Biochem..

[35] Wu K.K., Thiagarajan P. (1996). Role of endothelium in thrombosis and hemostasis. Annu. Rev. Med..

[36] Gryglewski R. (2008). Prostacyclin among prostanoids. Pharmacol. Rep..

[37] Inoue T., Kobayashi K., Inoguchi T., Sonoda N., Fujii M., Maeda Y. (2011). Reduced expression of adipose triglyceride lipase enhances tumor necrosis factor α-induced intercellular adhesion molecule-1 expression in human aortic endothelial cells via protein kinase C-dependent activation of nuclear factor-κB. J. Biol. Chem..

[38] Schrammel A., Mussbacher M., Wölkart G., Stessel H., Pail K., Winkler S. (2014). Endothelial dysfunction in adipose triglyceride lipase deficiency. Biochim. Biophys. Acta Mol. Cell Biol. Lipids.

[39] Jakubowski A., Sternak M., Jablonski K., Ciszek-Lenda M., Marcinkiewicz J., Chlopicki S. (2016). 1-Methylnicotinamide protects against liver injury induced by concanavalin A via a prostacyclin-dependent mechanism: a possible involvement of IL-4 and TNF-α. Int. Immunopharmacol..

[40] Bryniarski K., Biedron R., Jakubowski A., Chlopicki S., Marcinkiewicz J. (2008). Anti-inflammatory effect of 1-methylnicotinamide in contact hypersensitivity to oxazolone in mice; involvement of prostacyclin. Eur. J. Pharmacol..

[41] Chlopicki S., Swies J., Mogielnicki A., Buczko W., Bartus M., Lomnicka M. (2007). 1-Methylnicotinamide (MNA), a primary metabolite of nicotinamide, exerts anti-thrombotic activity mediated by a cyclooxygenase-2/prostacyclin pathway. Br. J. Pharmacol..

[42] Chlopicki S., Gryglewski R. (2005). Angiotensin converting enzyme (ACE) and HydroxyMethylGlutaryl-CoA (HMG-CoA) reductase inhibitors in the forefront of pharmacology of endothelium. Pharmacol. Rep..

