# Supplementary material for: Vascular lipid droplets formed in response to TNF, hypoxia, or OA: biochemical composition and prostacyclin generation
Source: J Lipid Res. 2023 Mar 17;64(5):100355. doi: 10.1016/j.jlr.2023.100355 (PMC10233209; doi:10.1016/j.jlr.2023.100355)
Supplement: Supplemental Figure S1 [file mmc1.docx]

**Vascular lipid droplets formed in response to TNF, hypoxia or OA: biochemical composition and prostacyclin generation**

Marta Z. Pacia^1*^, Natalia Chorazy^1^, Magdalena Sternak^1^, Kamila Wojnar-Lason^1,2^, Stefan Chlopicki^1,2^

**Corresponding autor:** [marta.pacia@jcet.eu](mailto:marta.pacia@jcet.eu)

^1^Jagiellonian Centre for Experimental Therapeutics (JCET), Jagiellonian University, 14

Bobrzynskiego Str., 30-348 Krakow, Poland

^2^Chair of Pharmacology, Jagiellonian University, 16 Grzegorzecka Str., 31-531 Krakow, Poland

**Keywords:** endothelium, inflammation, atglistatin, lipolysis, prostacyclin, Raman spectroscopy, fluorescence imaging, angiotensin II, lipid droplets, adipose triglyceride lipase (ATGL)


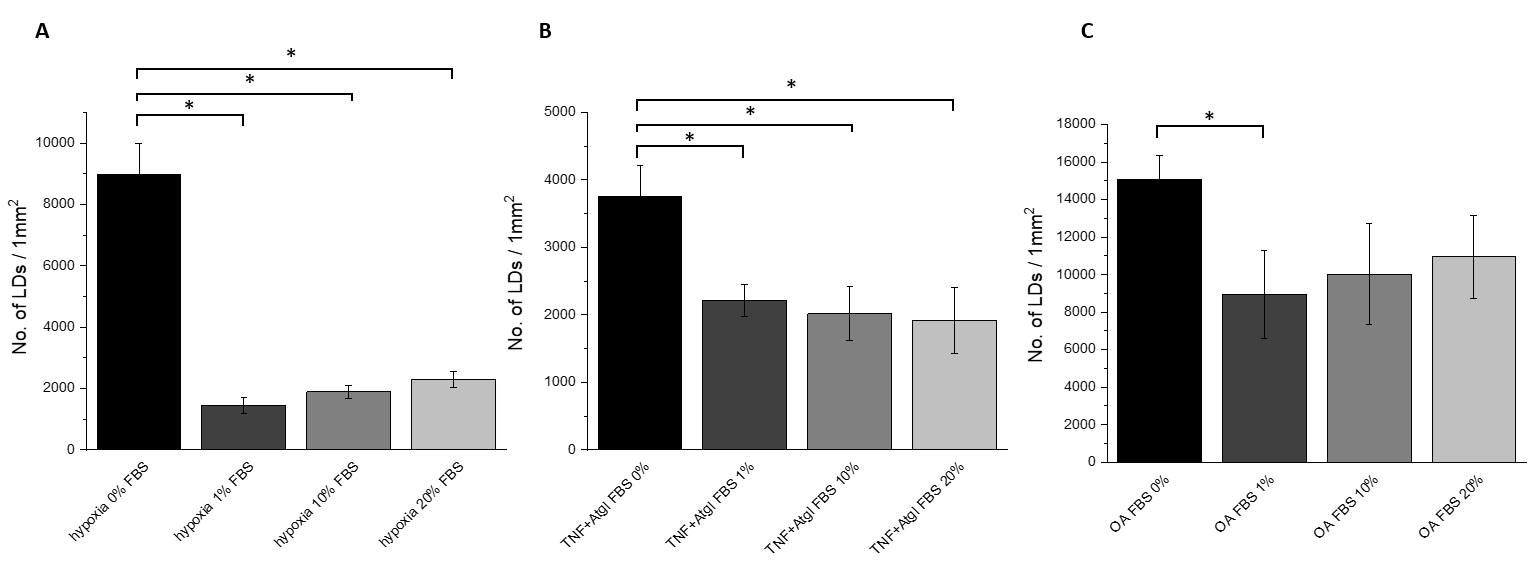


***Fig. S1.*** *Dependence of the number of LDs on the presence/concentrations of FBS in the medium under (A) hypoxic conditions, (B) under the influence of TNF, and (C) in the presence of oleic acid.*
